# Optical molecular imaging of lysyl oxidase activity – detection of active fibrogenesis in human lung tissue†
†Electronic supplementary information (ESI) available: Fig. S1, full experimental details and procedures, characterisation of compounds **1–11**. Detail information on human and asinine *ex vivo* tissue models, western blot, immunohistochemistry, fibered confocal fluorescence microscopy (FCFM). See DOI: 10.1039/c5sc01258a


**DOI:** 10.1039/c5sc01258a

**Published:** 2015-06-08

**Authors:** Tashfeen Aslam, Amy Miele, Sunay V. Chankeshwara, Alicia Megia-Fernandez, Chesney Michels, Ahsan R. Akram, Neil McDonald, Nik Hirani, Chris Haslett, Mark Bradley, Kevin Dhaliwal

**Affiliations:** a School of Chemistry , EaStChem , University of Edinburgh , Joseph Black Building, West Mains Road , Edinburgh , EH9 3FJ , UK . Email: mark.bradley@ed.ac.uk; b Pulmonary Optical Molecular Imaging Group , MRC/Centre of Inflammation Research , Queen's Medical Research Institute , University of Edinburgh , 47 Little France Crescent , EH16 4TJ , Edinburgh , UK . Email: Kev.Dhaliwal@ed.ac.uk

## Abstract

Aberrant fibrogenesis is a feature of many diseases in multiple organ systems. The lysyl oxidase family of enzymes are central to tissue homeostasis and elevated lysyl oxidase activity is implicated in fibroproliferation as well as in cancer stroma. We have synthesised a novel fluorogenic reporter for monitoring lysyl oxidase activity that generates a 3–5 fold increase in fluorescence following probe activation in ventilating fibrotic *ex vivo* asinine lung and *ex vivo* human lung tissue. The probe termed “oLOX” can provide real-time measurement of lysyl oxidase activity in a number of biological settings and is tractable from an *in vitro* setting to man.

## Abstract 2

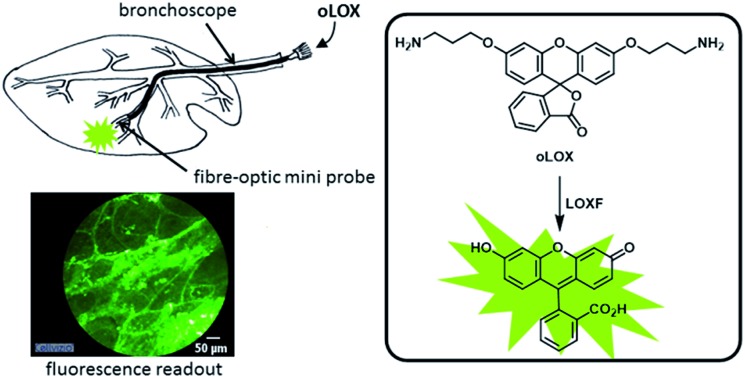
A fluorogenic probe provides real-time measurement of lysyl oxidase activity in *ex vivo* asinine and human lung tissue.

## Introduction

Aberrant fibrogenesis is a feature of many diseases in multiple organ systems and is characterised by tissue injury, remodelling and incomplete repair resulting in the excessive deposition of collagen.^1^,^2^ Fibrogenesis is a prominent feature of several lung diseases ranging from adult respiratory distress syndrome in intensive care to chronic obstructive pulmonary disease and interstitial lung diseases.^3^ The global impact of these diseases is huge, causing significant financial burden.^4^ Despite this, treatment options are very limited and monitoring of disease progression remains a challenge, as there are no rapid bedside biomarkers that can detect active fibroproliferation. Blood biomarkers offer poor molecular specificity for lung pathology, while pulmonary biopsy is invasive and fixed tissue cannot inform on the dynamic enzyme activity that exists *in vivo*.^5^

The Lysyl oxidases are copper dependent amine oxidases that play a central role in fibrogenesis.^6^ They facilitate the covalent cross-linking found within elastin and collagen by catalysing the oxidative deamination of peptidyl-lysine and hydroxylysine residues, a step that is crucial to the mature and functional extracellular matrix.^7^–^9^ There are five proteins within the lysyl oxidase family (LOXF): LOX, LOXL1 (lysyl oxidase like-one), LOXL2, LOXL3 and LOXL4, all of which share the same enzymatically active C-terminus and the cofactor lysine tyrosylquinone.^10^,^11^ A by-product of all of these enzymes is hydrogen peroxide. Lysyl oxidases are important in cancer progression and in many fibrotic diseases, including those affecting the lung.^12^–^15^ Of the 5 members of the LOXF, two in particular have been the basis of extensive investigation and therapeutic targeting: LOX and LOXL2. LOX expression correlates with poor survival and is a therapeutic target for patients with certain cancers,^14^ while LOXL2 is a focus in the monitoring and treatment of fibrotic lung disease.^15^,^16^

As the potential for lysyl oxidases as therapeutic targets becomes clear, there is a discernible need for the development of selective and sensitive oxidase substrates to monitor enzymatic activity in complex biological systems. As a result, fluorescence-based analysis methods have attracted much attention as they can provide simple, effective and powerful tools^17^ for real-time monitoring of LOX enzyme activities *in vitro* and *in vivo*.^18^–^20^ Until recently, such reporters have been designed either on a coumarin^21^,^22^ or resorufin^23^ scaffold and are based on a single amine oxidation/β-elimination resulting in an amplification of fluorescent signal. However, the excitation wavelength for coumarin (*λ*_ex_ 360 nm) limits its applicability for cellular-based imaging. Amplex red (*N*-acetyl-3,7-dihydroxyphenoxazine) is a non-fluorescent derivative of dihydroresorufin that is converted to fluorescent resorufin on reaction with hydrogen peroxidase.^24^,^25^ This reagent has been utilised in a number of settings including the quantification of neutrophil NADPH oxidase, monoamine oxidase (MAO), glucose oxidase and LOX.^19^,^24^,^25^ While providing a sensitive method for the detection of hydrogen peroxide,^26^ Amplex red is non-specific and clearly cannot be used *in vivo* or in cells.^19^

**Scheme 1:**
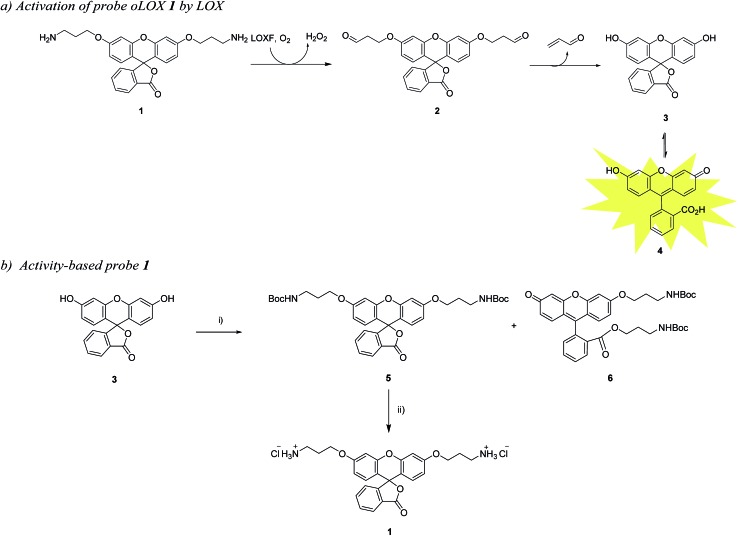
(a) Pathway for the fluorescence activation of probe oLOX **1** by LOX. (b) Synthetic route for oLOX, (i) Br(CH_2_)_3_NHBoc, Ag_2_O, MeCN, py, 4 Å MS, 40 °C, 48 h, **5** (15%) and **6** (65%); (ii) HCl, DCM/ether, 2 h, 93%.

In a recent publication, Li *et al.* reported a probe for the fluorometric detection of monoamine oxidases A and B *in vitro*. The probe was synthesised over 5 steps using the familiar and inexpensive dye, fluorescein **3**. While utilising the amine oxidation/β-elimination mechanism in the presence of MAO-A and B, the fluorescent reporter probe was methyl fluorescein, a dye with a lower quantum yield than **4**.^27^ In order to develop a probe for demonstrating the activity of LOXF *in vivo*, a reporter probe with higher quantum yield was required. Herein, we report the synthesis and *ex vivo* biological evaluation of an easily synthesised activity-based fluorescent probe for LOXF that utilises a fluorescein scaffold (Scheme 1).

**Scheme 2:**
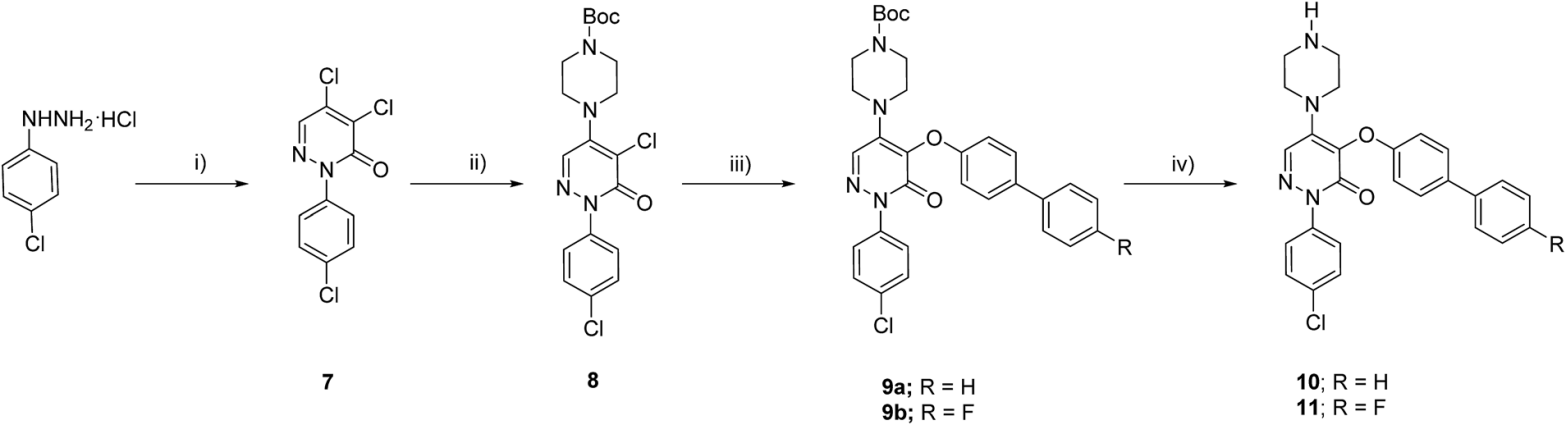
Synthetic route to the LOX inhibitors. (i) Mucochloric acid, 50% aq. MeOH, 3 h, 60%; (ii) *N*-Boc-piperazine, NaI, dioxane, 100 °C, 20 h, 70%; (iii) bisphenol derivative, CuI, Cs_2_CO_3_, BINOL, dioxane, reflux, 12 h, 58–73%; (iv) 20% TFA in DCM, 1 h, quantitative.

To validate the activation of the optical LOXF probe (oLOX) **1** in the presence of LOX, selective inhibition was desired. β-Aminopropionitrile (BAPN) has been extensively used as the reference LOX inhibitor, with a reported IC_50_ of 10 μM.^28^ Though BAPN is widely used as a specific irreversible inhibitor of LOX, it does have affinity for other oxidases,^29^,^30^ indeed its structure suggests non-specificity. Over the last two decades selective inhibitors of LOX, as anti-fibrotic agents, have been developed.^31^–^35^ Of these the pyridazinone-based class are among the most potent (IC_50_ 0.005–0.07 μM).^36^ Compounds **10** (IC_50_ 3 nM)^37^ and **11** were thus synthesized for application in inhibition studies (Scheme 2).

oLOX was evaluated for LOX activity in freshly isolated human lung tissue, showing for the first time, the ability to measure the dynamic activity of LOXF with optical detection in this model and the potential for *in vivo* utility.

## Results and discussion

### Synthesis of optical LOXF probes

The chemical “masking” of the phenol groups of fluorescein can suppress fluorescence and facilitate cell permeability.^38^,^39^ In this investigation, both phenolic groups of **3** were selectively alkylated with aminopropyl moieties, it being hypothesised that upon treatment of oLOX **1** with the appropriate oxidases, the two amino groups would be oxidised to give the bis-aldehyde **2** followed by dual β-elimination to release acrolein and fluorescein **4** (Scheme 1a).

The synthesis of oLOX **1** was complicated due to the nature of xanthene dyes such as fluorescein, which exists in either of the two forms, the quinoid **4** and/or lactone **3** form, depending on the pH and solvent environment.^40^ The fluorescent, xanthen-3-one tautomer form, is favoured in non-acidic aqueous solutions; whereas a non-fluorescent, lactone tautomer **3**, is favoured at acidic pH's or in non-aqueous solvents. Typically alkylation of fluorescein gives the dual ether/ester (**6**) as the major product, rather than the bis-ether (**5**). To develop the optimal conditions to synthesise the desired bis-ether product **1**, reactions conditions were optimised looking at various solvents and bases.^41^ It is known that the use of heterogeneous Ag(i) salts favours the desired phenol alkylation reaction over ester formation, and the use of silver oxide in acetonitrile^42^ with molecular sieves was effective for the synthesis of **5** in moderate yield (Scheme 1b), with the crude reaction mixture affording **5** and **6** in 15% and 65% yields, respectively. **5** was non-fluorescent while quinoid **6** showed emission characteristics (*λ*_max_ = 520 nm) of fluorescein. Treatment of **5** with hydrochloric acid in DCM/ether gave reporter **1** as its HCl salt in quantitative yield. As anticipated, the final probe had good aqueous solubility and was non-fluorescent.

The two specific and potent LOX inhibitors **10** and **11** were synthesised (Scheme 2). **11** was designed based on structure activity relationship of similar inhibitors where the introduction of 4-(*p*-fluorophenyl)phenol significantly enhanced the potency of the inhibitor.^32^ 2-Phenyl-3(2*H*)-pyridazinones, were synthesised in two steps by condensation of mucochloric acid with aryl hydrazine to afford the dichloro pyrazone derivative **7**. In the next step, nucleophilic substitution with *N*-Boc piperazine derivative afforded **8**. Reaction of **8** with 4-phenylphenol or 4-(*p*-fluorophenyl)phenol and Boc deprotection afforded the inhibitors **10** and **11** in good yields (50–55%).

### LOX and LOXL2 are expressed in human lung tissue

LOX is important in lung development and is expressed in normal lung tissue.^43^,^44^ Pathological activity of LOXL2 has been shown by Barry-Hamilton *et al.* with increased expression of LOXL2 in the lung tissue of patients with idiopathic pulmonary fibrosis (IPF),^15^ while Chien *et al.* found an association between serum LOXL2 levels and the progression of IPF.^16^ Increased expression of LOX has also been well demonstrated in experimental models of pulmonary fibrosis.^45^,^46^ Hence we wished in particular to begin evaluating our probe in the setting of human lung pathology.

**Fig. 1:**
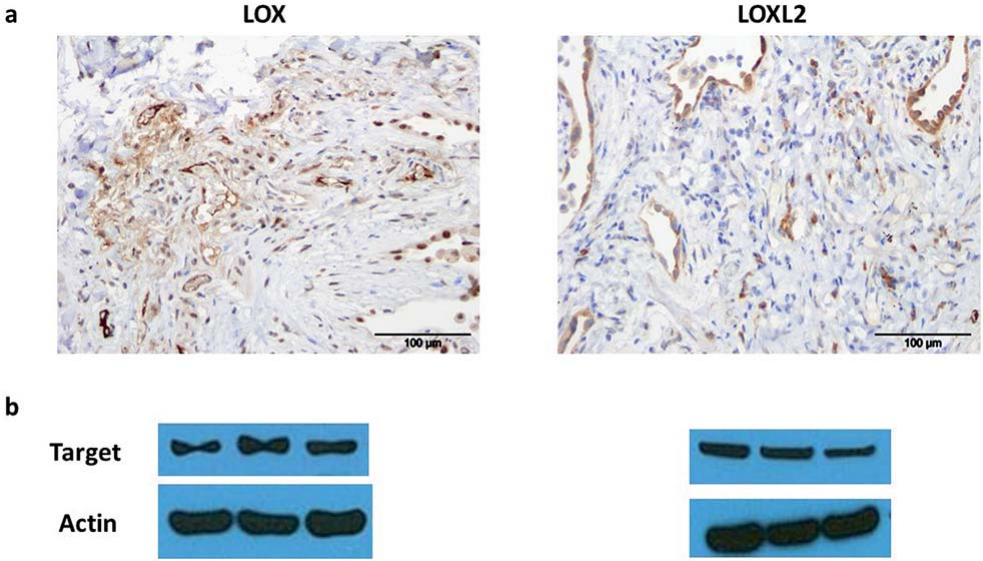
LOX and LOXL2 are expressed in aged human lung tissue homogenates. (a) Immunohistochemical analysis and (b) western blot of LOX and LOXL2 expression in aged human (55–81 years) lung tissue. Strongest bands for LOX were at 50 kDa, consistent with the glycosylated pro-lysyl oxidase, but mature 32 kDa LOX was also present in several samples (ESI, Fig. S1†). Data representative of a minimum of 3 experimental replicates. Scale-bar = 100 μm.

The expression of LOX and LOXL2 was firstly confirmed in aged human (55–81 years) lung tissue homogenates using western blot and immunohistochemistry. The 50 kDa pro-LOX is secreted from cells then cleaved to the 32 kDa mature protein by bone morphogenetic protein-1.^10^ In this study we demonstrated the presence of the 50 kDa pro-LOX protein and the active 32 kDa LOX protein in human lung tissue as well as the enzymatically active 63 kDa LOXL2 (Fig. 1). Immunohistochemical data supported previous findings whereby LOX and LOXL2 were found to be expressed in lung tissue.^15^ Since LOX and LOXL2 were expressed in human lung tissue, homogenised lung tissue was deemed an appropriate model to evaluate the utility of the activity-based sensor oLOX **1** (Scheme 1).

### oLOXF detects increased LOXF activity in aged human tissue and is inhibited by selective inhibitors

**Fig. 2:**
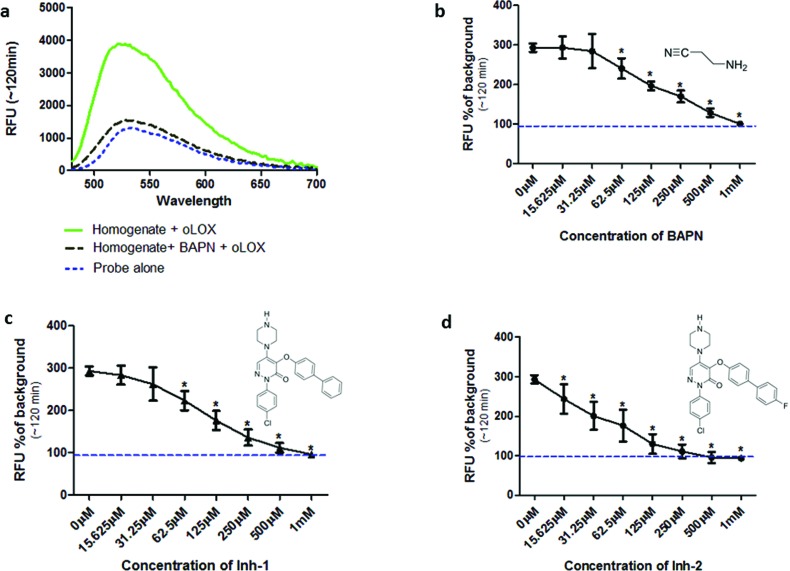
oLOX is activated in the presence of aged human lung tissue homogenate as shown by an inhibitable increase in fluorescence. (a) Fluorescent intensity of 10 μM oLOX after 2 h incubation with aged human lung tissue homogenate (solid line) ± 500 μM BAPN. Spectra were recorded with *λ*_ex_ = 450 nm. (b–d) Human lung homogenate was pre-incubated with dilutions of inhibitors at 37 °C for 1 h prior to the addition of 10 μM oLOX. Each graph shows the resultant fluorescence presented as percentage above background after 1 h incubation of the reaction (representative of *n* = 3). The minimum concentration of BAPN (b) and Inh-1 (c) found to have a significant inhibitory effect was 62 μM (*p* < 0.01, one way ANOVA), whereas the minimum concentration of Inh-2 (d) found to have a significant inhibitory effect was 15 μM (*p* < 0.01, one way ANOVA). The dotted line represents background fluorescence of oLOX, error bars depict standard deviation.

Incubation of oLOX with lung tissue homogenate resulted in up to a 300% increase in fluorescence (Fig. 2a). The temporal kinetics of oLOX activation with human tissue at the concentrations used in this study showed significant fluorescence amplification after 30 min (*p* < 0.01) and plateaued between 50 min and 2 h (ESI Fig. S2†). Importantly, oLOX activation was completely inhibited in a concentration dependent manner by pre-incubation of the homogenate with BAPN (Fig. 2b) and the inhibitors **10** and **11** (Fig. 2c and d). As predicted inhibitor **11** was a more potent inhibitor of probe activation (Fig. 2d). BAPN is a time and temperature dependant^47^ irreversible inhibitor of LOX.^48^,^49^ BAPN has been shown to inhibit LOXL1,^50^ LOXL3 51 and LOXL4,^52^ but inhibition of LOXL2 by BAPN is more controversial with mixed reports of success.^26^,^53^ We have shown that both active LOX and LOXL2 are present within the lung tissue homogenates and hypothesise that each of these enzymes contribute to the increase in fluorescent signal observed with our probe. We have not investigated the expression of other members of the LOXF within the lung tissue and it is certainly possible that they are present at low levels, contributing to the fluorescent signal.

**Fig. 3:**
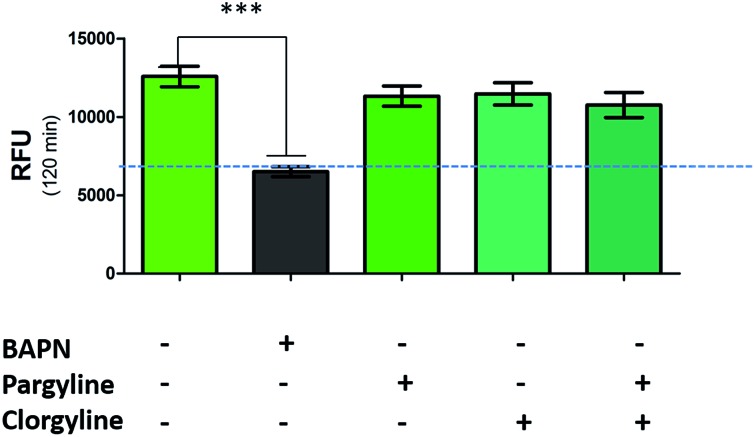
oLOX is not activated by monoamine oxidases. Human lung homogenate was pre-incubated (in triplicate) with 500 μM of BAPN, clorgyline and/or pargyline at 37 °C for 1 h prior to the addition of 10 μM oLOX. The bar show the mean resultant fluorescence intensity after 2 h incubations (*n* = 3). ***p < 0.001, one way ANOVA, dotted line represents background fluorescence of oLOX, error bars depict standard error of the mean.

While a probe of related structure has previously been used to report on levels of MAO,^27^ the potential contribution of MAO to oLOX activation in human lung tissue was negated by the fact that clorgyline (a MAO-A inhibitor) and pargyline (a MAO-B inhibitor) did not cause any significant reduction in fluorescent signal (Fig. 3). Furthermore, BAPN does not inhibit MAO,^54^ thus confirming that MAO does not cleave **1** in this model.

### Optical molecular imaging of LOX

Whilst fluorometric determination of LOX in human tissue has been demonstrated (Fig. 2), the pivotal advance would be the dynamic imaging of LOX activity in tissue *in situ* without the need for homogenisation. This heralds the capability to perform optical molecular ‘biopsies’ *in situ* with optical molecular imaging techniques.^55^,^56^ Fibred confocal fluorescence microscopy (FCFM) allows a flexible optical fibre bundle to be passed down the working channel of an endoscope while utilising laser excitation at 488 nm. FCFM has been successfully utilised in a number of organ systems including the pulmonary tract,^57^,^58^ where the autofluorescence of the elastin allows visualisation of tissue architecture. This approach allows the real-time *in vivo* visualisation of tissue at a cellular level and is a clinically applicable strategy.

**Fig. 4:**
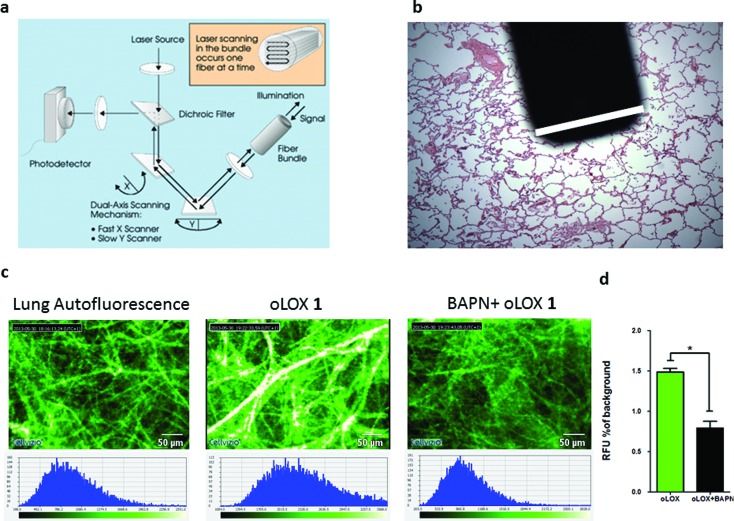
oLOX can be used alongside fibred confocal fluorescence microscopy (FCFM) to demonstrate oLOX activity in *ex vivo* human lung tissue biopsies. (a) Architecture of the FCFM (Cellvizio®-Lung System (Mauna Kea Technologies, Paris, France))† involving a fibre bundle and scanning confocal microscopy (b) the tip of the fibre optic is superimposed on a fixed lung tissue section with the white bar corresponding to the diameter of the fibre (1.4 mm). (c) Representative single frame images generated by FCFM on fresh sections of aged human lung tissue following incubation with 10 μM oLOX. The graph (d) represents the mean auto-fluorescence of each 60 sec video imaging period adjusted for tissue auto-fluorescence (*n* = 4). Note the significant reduction in fluorescence on incubation with the lysyl oxidase inhibitor, BAPN (*p = 0.0286, Wilcoxon signed rank test).

Hence we evaluated oLOX **1** in conjunction with FCFM, as an optical molecular imaging strategy by applying oLOX to aged human lung tissue *ex vivo*. To demonstrate that the resultant increase in fluorescence detected by FCFM could be inhibited and prove the specificity of oLOX, we also incubated human lung tissue with BAPN (Fig. 4).

**Fig. 5:**
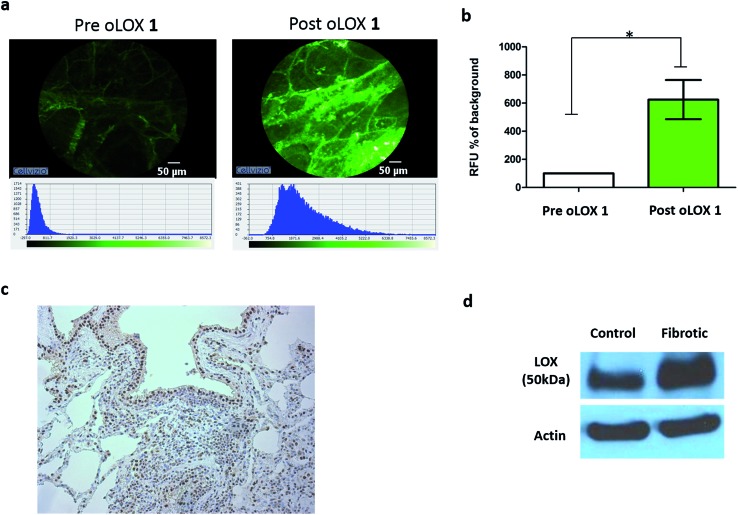
oLOX can be used to image LOXF activity in a fibrotic ventilating *ex vivo* asinine lung model. Catheter installation of 200 μM oLOX instilled in a total volume of 1 mL in PBS into a whole fibrotic ventilating *ex vivo* asinine lung results in a significant increase in fluorescent signal (a and b, *p = 0.0313, Wilcoxon signed rank test, *n* = 3); (c) tissue shown in (a) was excised and evaluated for LOX expression using immunohistochemistry (20× lens). (d) Western blot confirmed expression of LOX in both fibrotic and grossly unaffected (control) areas of *ex vivo* asinine lung tissue.

Moving from sections of tissue (Fig. 4) to whole organ imaging was the next challenge. Hence, in order to assess the utility of oLOX **1***in situ*, a ventilating *ex vivo* asinine lung model was used (Fig. 5 and ESI Fig. S3–4†). We have recently reported that aged donkeys suffer from a high prevalence of pulmonary fibrosis (35%) (often resulting in euthanasia), which has been likened to a fibrotic interstitial lung disease of humans.^59^ A whole, *ex vivo*, donkey lung was thus used to assess the utility of combining FCFM with the local intrapulmonary delivery of oLOX. Utilising this size relevant and spontaneous model negated the need for an experimental model under the 3Rs (Replacement, Reduction and Refinement) ethos. A significant increase in fluorescent signal over background was detected following the installation of oLOX **1** into fibrotic regions of whole ventilating *ex vivo* asinine lung that were subsequently confirmed to express LOX (Fig. 5c). The coupling of oLOX **1** with FCFM enables the minimally invasive visualisation of temporal and spatial alterations in the molecular activity of LOXF.

## Conclusions

We have successfully designed and synthesized an activity-based fluorescent probe capable of the real-time quantification of LOXF activity in fibrogenic conditions. The activation of the probe by LOXF was confirmed in human lung homogenate models. Furthermore, probe activation was inhibited in the presence of a specific LOX inhibitor, thus confirming target specificity. The probe also showed utility in a size-relevant model of lung fibrogenesis. This optical Smartprobe has the potential to image real-time LOXF activity within the lungs of patients. Significant signal amplification is detected after 30 min, a time-point that is feasible for bedside imaging. Whilst we have not yet assessed the specificity of oLOX to individual members of the LOXF family, targeting individual enzymes is a future goal, exploiting the use of specific inhibitor based imaging agents. A further goal is to develop oLOX sensors that permit more rapid fluorescent amplification.

## Supplementary Material

Supplementary movie

Supplementary information

Supplementary information
