# Supplementary material for: Optical molecular imaging of lysyl oxidase activity – detection of active fibrogenesis in human lung tissue
Source: Chem Sci. 2015 Jun 8;6(8):4946–53. doi: 10.1039/c5sc01258a (PMC6088439; doi:10.1039/c5sc01258a)
Supplement: Supplementary file 2 [file SC-006-C5SC01258A-s002.pdf]

Electronic Supplementary Material (ESI) for Chemical Science.
This journal is © The Royal Society of Chemistry 2015

ESI Figure S4: oLOX instillation into a ventilating *ex vivo* fibrotic asinine lung alongside FCFM. Video of oLOX instillation into a ventilating *ex vivo* asinine lung model. Lungs were connected via a cuffed endotracheal tube to a positive pressure mechanical ventilator. A video endoscope was used to navigate to a pre-determined region of interest within the *ex vivo* lung. The endoscope was secured within a bronchus before the miniprobe (ProFlexTM S-1500, Mauna Kea Technologies, Paris, France) was passed down the working channel of the endoscope and advanced into the alveolar space. 200µM oLOX was then instilled in a total volume of 1ml in PBS.

Keywords: FCFM, ex vivo, asinine lung, LOX probe, oLOX
