# Supplementary material for: Optical molecular imaging of lysyl oxidase activity – detection of active fibrogenesis in human lung tissue
Source: Chem Sci. 2015 Jun 8;6(8):4946–53. doi: 10.1039/c5sc01258a (PMC6088439; doi:10.1039/c5sc01258a)
Supplement: Supplementary file 3 [file SC-006-C5SC01258A-s003.pdf]

Electronic Supplementary Material (ESI) for Chemical Science.
This journal is © The Royal Society of Chemistry 2015

Optical molecular imaging of lysyl oxidase activity – detection of active fibrogenesis in human lung tissue

Tashfeen Aslam,[a#] Amy Miele,[b#] Sunay V. Chankeshwara,[a#] Alicia Megia-Fernandez,[a] Chesney Michels,[b] Ahsan Akram,[b] Neil McDonald,[b] Nik Hirani,[b] Chris Haslett,[b] Mark Bradley,[a] & Kevin Dhaliwal.[b*]*

[a]School of Chemistry, EaStChem, University of Edinburgh, Joseph Black Building, West Mains Road, Edinburgh, EH9 3FJ, UK
[b]Pulmonary Optical Molecular Imaging Group, MRC/Centre of Inflammation Research, Queen's Medical Research Institute, University of Edinburgh, 47 Little France Crescent, EH16 4TJ, Edinburgh, UK

E-mail: Mark.Bradley@ed.ac.uk & Kev.Dhaliwal@ed.ac.uk

SUPPORTING INFORMATION

Table of contents

1. Experimental 2
1.1 General methods 2
1.2 Synthesis of oLOX probe (1) 3
1.3 Synthesis of LOX inhibitors 10 (Inh-1) and 11 (Inh-2) 4
1.4. Human and asinine *ex vivo* tissue models 7
1.5. Western Blots 8
1.6. Immunohistochemistry 8
1.7. Fibre confocal fluorescence microscopy (FCFM) 8
2. Supplementary Figures 9

1. Experimental

1.1 General methods

Reactions involving moisture sensitive reagents were performed under a positive pressure of dry nitrogen, and the glassware used was oven dried and cooled under dry nitrogen prior to the experiment. Reactions involving light sensitive compounds were kept wrapped in aluminium foil at all times. Thin-layer chromatography was performed by using aluminium sheet pre-coated with silica gel containing F_{254} phosphor and visualised by UV-illumination (λ = 254nm and 344nm) and/or stained with phosphomolybdic acid. Flash column chromatography was performed using glass column prepared with silica gel 60 (mesh 0.040-0.063). NMR spectra were recorded on automated

AV500 Bruker (500MHz) instrument in the indicated deuterated solvents at 298 K. ^{1}H spectra were referenced to the residual non-deuterated solvent peak and ^{13}C NMR to the deuterated solvent. Analytical reverse-phase high-performance liquid chromatography (RP–HPLC) was performed on an Agilent 1100 system equipped with a Discovery C18 reverse-phase column (5 cm x 4.6 mm, 5µm) with a flow rate of 1 mL/min and eluting with H_2O/MeOH/HCOOH (95/5/0.05) to H_2O/MeOH/HCOOH (5/95/0.05), over 6 min, holding at 95% MeOH for 4 min, with detection at 254 and 495nm and by evaporative light scattering. Electrospray ionization mass spectrometry (ESI–MS) analyses were carried out on an Agilent Technologies LC/MSD Series 1100 quadrupole mass spectrometer (QMS) in ESI mode. HRMS was obtained from the Mass Spectrometry department of the University of Edinburgh performed on a Finnigan MAT 900 XLP high resolution double-focussing mass spectrometer.

1.2 Synthesis of oLOX probe (1):

Di-*tert*-butyl[(3-oxo-3*H*-spiro[2-benzofuran-1,9'-xanthene]-3',6'-diyl)bis(oxypropane-3,1-diyl)]biscarbamate (5) and 3-(*tert*-butoxycarbonyl)propyl 2-(3-(3-(*tert*-butoxycarbonyl)propoxy)-6-oxo-6*H*-xanthen-9-yl)benzoate (6): Fluorescein **3** (116 mg, 0.34 mmol) was disolved in anhydrous acetonitrile (8mL) under nitrogen in the presence of 4 Å molecular sieves. Silver(I) oxide (248 mg, 1.10 mmol), (boc-amino)propyl bromide (250 mg, 1.1 mmol) and 2 drops of pyridine were added to the reaction mixture. The reaction mixture was stirred at 40 °C for 48 hours. Thereafter, the reaction solution was filtered through Celite® and was concentrated. Diethyl ether (20 mL) was added to the residue to triturate the unwanted dialkylated by-product. The suspension was filtered and the filtrate was concentrated to give an oily residue. The resultant was then purified by flash column chromatography (silica gel, eluting with hexane/EtOAc from 10:1 to 3:1 (v/v)) to give compound **5** as a colourless oil (32 mg, 15%) and compound **6** as an orange solid (140 mg, 55%).

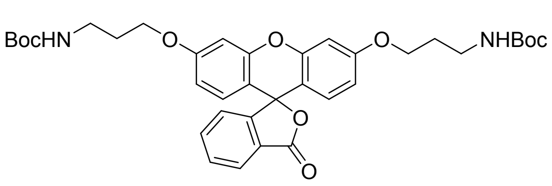

5

Compound **5**: **TLC:** R_f = 0.43 (1:1 hexane/EtOAc); **^{1}H NMR:** (500 MHz, $CDCl_3$) 8.01 (1H, d, *J* 7.5, CH), 7.65 (1H, t, *J* 7.4, CH), 7.61 (1H, t, *J* 7.4, CH), 7.14 (1H, d, *J* 7.5, CH), 6.74 (2H, d, *J* 2.4, 2x CH), 6.67 (2H, d, *J* 8.8, 2x CH), 6.59 (2H, dd, *J* 2.4, 8.8, 2x CH), 4.75 (2H, broad s, NH), 4.04 (4H, t, *J* 6.0, 2x CH_2), 3.34-3.28 (4H, m, 2x CH_2), 2.02-1.95 (4H, m, 2x CH_2); **^{13}C NMR:** (125 MHz, $CDCl_3$) 169.49 (C=O), 160.50 (2x C), 156.02 (2x C=O), 153.20 (C), 152.46 (2x C),134.99 (CH), 129.69 (CH), 129.11 (2x CH), 126.82 (C), 125.02 (CH), 123.91 (CH), 111.97 (2x CH), 111.36 (2x C), 101.42 (2x CH), 83.18 (C), 79.63 (C), 79.32 (C), 66.09 & 65.88 (CH_2), 37.88 (2x CH_2), 31.90 (2x CH_2), 28.38 (CH_3); **IR:** 3355.5, 2975.6, 2931.8, 1764.4, 1695.9, 1504.1, 1248.4, 1174; **ESIMS** (*m*/*z*): 647.2 $[M+H]^+$.

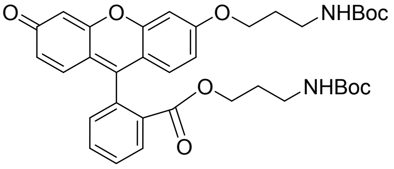

6

Compound **6**: **TLC:** R_f = 0.05 (1:1 hexane/EtOAc); m.p. 138-140 °C; **^{1}H NMR:** (500MHz, $CDCl_3$) 8.01 (1H, d, *J* 7.5, CH), 7.65 (1H, t, *J* 7.4, CH), 7.61 (1H, t, *J* 7.4, CH), 7.14 (1H, d, *J* 7.5, CH), 6.74 (2H, d, *J* 2.4, 2x CH), 6.67 (2H, d, *J* 8.8, 2x CH), 6.59 (2H, dd, *J* 2.4, 8.8, 2x CH), 4.75 (2H, broad s, NH), 4.04 (4H, t, *J* 6.0, 2x CH_2), 3.34-3.28 (4H, m, 2x CH_2), 2.02-1.95 (4H, m, 2x CH_2); **^{13}C NMR:** (500 MHz, $CDCl_3$) 169.46 (C=O), 160.50 (2x C), 156.02 (2x C=O), 153.20 (C), 152.46 (2x C),134.99 (CH), 129.69 (CH), 129.11 (2x CH), 126.82 (C), 125.02 (CH), 123.91 (CH), 111.97 (2x CH), 111.36 (2x C), 101.42 (2x CH), 83.18 (C), 79.63 (C), 79.32 (C), 66.09 & 65.88 (CH_2), 37.88 (2x CH_2), 31.90 (2x

CH_2), 28.38 (CH_3); **IR:** 3325.9, 2972.7, 2928.4, 1707.4, 1642.6, 1509.6, 1251.6, 1168.98; **ESIMS** (*m*/*z*): 647.2 $[M+H]^+$.

3',6'-Bis(3-aminopropoxy)-3*H*-spiro[2-benzofuran-1,9'-xanthen]-3-one dihydrochloride (1): The

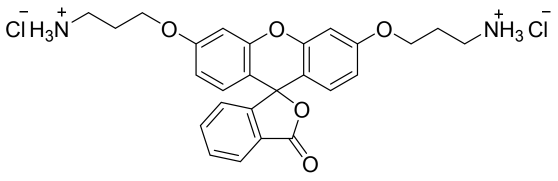

1

protected amine **5** (21 mg, 0.03 mmol) was dissolved in 10% HCl in dichloromethane and diethyl ether (1:1, 4 mL) and the resulting mixture was stirred for 2 hours. The solvent was removed *in vacuo* to afford the product **1** as an off-white solid (15.8 mg, 93%). **^{1}H NMR:** (500 MHz, D_2O) 7.99 (1H, d, *J* 7.5, CH), 7.73 (1H, t, *J* 7.4, CH), 7.68 (1H, t, *J* 7.4, CH), 7.14 (1H, d, *J* 7.5, CH), 6.77 (2H, d, *J* 2.2, 2x CH), 6.73 (2H, d, *J* 8.9, 2x CH), 6.63 (2H, dd, *J* 2.2, 8.9, 2x CH), 4.07 (4H, t, *J* 5.7, 2x CH_2), 3.14 (4H, t *J* 7.2, 2x CH_2), 2.09 (4H, quintet, *J* 6.5, 2x CH_2); **^{13}C NMR:** (125 MHz, D_2O) 172.10 (C=O), 160.37 (C), 152.47 (C), 151.72 (C), 136.14 (CH), 130.46 (CH), 129.30 (CH), 126.06 (C), 125.19 (CH), 124.16 (CH), 112.32 (CH), 110.89 (C), 101.53 (CH), 88.13 (C), 65.88 (CH_2), 37.32 (CH_2), 26.20 (CH_2); **IR:** 3279.3, 1633.8; **ELSD:** t_R = 4.72 min; **ESIMS** (*m*/*z*): 447.2 $[M+H]^+$.

1.3 Synthesis of LOX inhibitors 10 (Inh-1) and 11 (Inh-2):

4,5-Dichloro-2-(4-chlorophenyl)-2,3-dihydropyridazin-3-one (7):

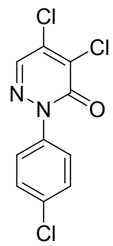

Mucochloric acid (4.2 g, 25mmol, 1 eq) was stirred in 50% aqueous MeOH (20 mL) To this suspension, a mixture of 4-chlorophenyl hydrazine hydrochloride (5.8 g, 33 mmol, 1.3 eq) in 50% aqueous MeOH (20 mL) was added dropwise, and the resultant mixture was stirred at room temperature. After 3h, the orange precipitate was collected, washed with 50% aqueous MeOH (20 mL). After drying under suction for 30 min, the solid was dissolved in acetic acid (30 mL) and stirred at reflux for 30 min. After cooling the reaction mixture to room temperature, it was poured into water (150 mL) and cooled in an ice bath. The precipitate was collected and washed with water (50 mL) and dried. The resultant solid was purified by recrystallisation from 90% aqueous EtOH (160 mL) to afford solid **7** (60%). **^{1}H NMR:** (500 MHz, $CDCl_3$): 7.92 (1H, s, CH), 7.56 (2H, d, *J* 9.0, CH), 7.45 (2H, d, *J* 9.0, CH); **^{13}C NMR:** (125 MHz, $CDCl_3$) 156.3 (C=O), 139.5 (C), 136.7 (CH), 136.6 (C), 135.7 (C), 134.9 (C), 129.3 (CH), 126.6 (CH); **ESIMS (*m/z*):** 275.1 $[M+H]^+$

***tert*-Butyl 4-[5-chloro-1-(4-chlorophenyl)-6-oxo-1,6-dihydropyridazin-4-yl]piperazine-1-carboxylate (8):** 4,5-Dichloro-2-(4-chlorophenyl)-2,3-dihydropyridazin-3-one (1 g, 3 mmol, 1 eq)

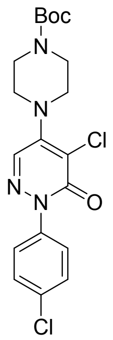

was suspended in dioxane (100 mL) followed by the addition of *N*-Boc-piperazine (1.5 g, 8 mmol, 2.7 eq) and sodium iodide (20 mg, 0.15 mmol, 0.02%) and the reaction mixture was stirred at 100 °C. After 20h, the volatiles are removed under vacuum and the residue was partitioned between DCM (50 mL) and water (20 mL). The organic layer was washed with brine (2 × 20 mL), dried (anhydrous $MgSO_4$) and evaporated under vacuum. The residue was suspended in Et_2O (20 mL) and stirred for 1h. The white solid was collected by filtration and washed again with Et_2O (20 mL) to afford white solid **8** (70%). **^{1}H NMR:** (500 MHz, $CDCl_3$): 7.76 (1H, s, CH), 7.59 (2H, d, *J* 8.83, CH), 7.45 (2H, d, *J* 8.83, CH), 3.65 (4H, t, *J* 5.0, CH), 3.46 (4H, t, *J* 5.0, CH), 1.52 (9H, s, CH); **ESIMS (*m/z*):** 425.1 $[M+H]^+$

4-(4-Fluorophenyl)phenol: A mixture of 4-bromophenol (1.73 g, 10 mmol, 1 eq), 4-fluorphenyl boronic acid (1.5g, 10.5 mmol, 1.1eq), potassium carbonate (4.2g, 30 mmol, 3eq), palladium acetate (0.12g, 0.5mmol, 5%) and triphenyl phosphine (0.6g, 0.5mml, 20%) in dioxane:water (9:1, 20 mL) was stirred at 100 °C. Upon completion of the reaction (TLC, 12h), the reaction mixture was evaporated to dryness and the residue was acidified with 1M HCl to pH 3. The aqueous layer was extracted with EtOAc (50 mL). The organic layer was washed with water (20 mL), brine (2 × 20 mL), dried (anhydrous $MgSO_4$) and evaporated under vacuum. The crude product was purified by flash column chromatography on silica gel eluting with 10% EtOAc in hexane to afford the product as a white solid (1.4 g, 74%). **^{1}H NMR:** (500 MHz, $CDCl_3$): 7.48 (2H, dd, *J* 8.5, CH), 7.42 (2H, d, *J* 8.5, CH), 7.10 (2H, t, *J* 8.5, CH), 6.89 (2H, d, *J* 8.5, CH), 4.74 (1H, bs, OH); **^{13}C NMR:** (125 MHz, $CDCl_3$) 162.8 (C), 160.9 (C), 154.7 (C), 136.6 (C), 132.6 (CH), 128.0 (CH), 127.9 (CH), 115.4 (CH), 115.2 (CH); **ESIMS (*m/z*):** 189.1 $[M+H]^+$

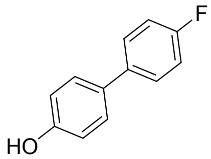

Compound 9a / 9b: Compound **8** (100 mg, 0.25 mmol, 1 eq), CuI (0.05 mmol, 0.2 eq), Cs_2CO_3 (0.50 mmol, 2 eq), BINOL (0.50 mmol, 2 eq) and 4-Phenyl-phenol for (**a**) or 4-fluorophenyl-4'-OH benzene for (**b**) (1.1 eq), were mixed and dissolved in dioxane (3 mL), and heated at 120^{0}C for 24 h. After cooling the mixture was poured into water (15 mL) and extracted with EtOAc (2x15 mL). The organic layers were combined and washed with brine (15 mL). The organic phase was dried (anhydrous Na_2SO_4) and purified by column chromatography: (silica gel, DCM-MeOH 100:0 to 250:1).

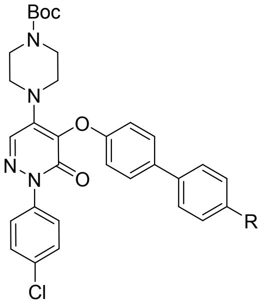

9a, R = H
9b, R = F

Compound **9a**: colourless oil (76 mg, 58%). **^{1}H NMR:** (500 MHz, $CDCl_3$) 7.86 (1H, s), 7.64 (2H, d, *J* 8.9), 7.56-7.52 (4H, m), 7.45-7.40 (4H, m), 7.34 (1H, t, *J* 7.4), 7.05 (2H, d, *J* 8.8), 3.50 (8H, s), 1.48 (s, 9H); **^{13}C NMR:** (125 MHz, $CDCl_3$) 157.30, 155.53, 154.45, 140.64, 140.53, 139.59, 136.19, 133.44, 133.12, 132.52, 128.75, 128.65, 128.52, 126.99, 126.93, 126.22, 115.69, 80.41, 48.51, 28.38. **ESIMS (*m/z*):** 559.3 $[M+H]^+$

Compound **9b**: (16 mg, 14%; also 81 mg obtained as an inseparable mixture with **9b** and starting material **8**, 73%. This mixture was used directly in the next step.) **^{1}H NMR:** (500 MHz, $CDCl_3$) 7.86 (1H, s), 7.63 (2H, d, *J* 8.9), 7.51-7.47 (4H, m), 7.41 (2H, d, *J* 8.9), 7.12 (2H, t, *J* 8.7), 7.04 (2H, d, *J* 8.8), 3.50 (8H, br s), 1.48 (s, 9H); **^{13}C NMR:** (125 MHz, $CDCl_3$) 163.26, 161.30, 157.24, 155.53, 154.45, 140.65, 139.56, 136.66, 135.24, 133.46, 133.10, 132.50, 131.47, 128.66, 128.47, 128.41, 127.51, 126.20, 124.22, 124.06, 115.75, 115.69, 115.52, 80.43, 48.52, 28.38. **ESIMS (*m/z*):** 577.2 $[M+H]^+$

Compound 10 (Inh-1): Compound **9a** (0.1 mmol) was dissolved in 20% TFA-DCM (5 mL) and stirred for 2h. After that time the solvent was removed under vacuum, the compound dissolved in DCM (20 mL), washed with $NaHCO_3$ sat (20 mL) and water (20 mL). The organic phase was dried over anhydrous Na_2SO_4, and concentrated under vacuum to afford **Inh-1** (**10**): quantitative; **TLC:** $R_f = 0.40$ (5:1 DCM/MeOH); **RP-HPLC** t_r 4.76 min; **^{1}H NMR:** (500 MHz, $CDCl_3$) 7.86 (1H, s), 7.64 (2H, d, *J* 8.9), 7.56-7.52 (4H, m), 7.45-7.39 (4H, m), 7.33 (1H, t, *J* 7.4), 7.05 (2H, d, *J* 8.8), 3.54 (4H, t, *J* 4.8), 2.96 (4H, t, *J* 4.7), 1.99 (br s, 1H); **^{13}C NMR:** (125 MHz, $CDCl_3$) 157.35, 155.62, 140.79, 140.58, 139.64, 136.09, 133.37, 132.84, 132.56, 128.74, 128.63, 128.47, 126.95, 126.92, 126.32, 115.71, 49.34, 45.81; **IR:** ν (cm^{-1}) 3297, 2924, 1640, 1609, 1488, 1259, 1214, 832, 761; **HRMS:** $[M+H]^+$ calculated 459.1582, found 459.1598.

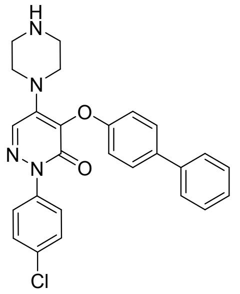

Compound 11 (Inh-2): 9b (mixture) (0.1 mmol) was dissolved in 20%TFA-DCM (5 mL) and stirredfor 2h. After that time solvent was removed under vacuum, the compound dissolved in DCM (20 mL), washed with $NaHCO_3$ sat (20 mL) and water (20 mL). The organic phase was dried over anhydrous Na_2SO_4, and concentrated under vacuum to afford **Inh-2** (**11**): purified by column chromatography (silica eluting with 5:1 DCM/MeOH), 56%; **TLC:** $R_f = 0.38$ (5:1 DCM/MeOH); **RP-HPLC** t_r 4.74 min; **^{1}H NMR:** (500 MHz, $CDCl_3$) 7.83 (1H, s), 7.60 (2H, d, *J* 9.0), 7.48-7.44 (4H, m), 7.38 (2H, d, *J* 8.9), 7.09 (2H, t, *J* 8.7), 7.01 (2H, d, *J* 8.7), 3.55 (4H, t, *J* 4.9), 2.98 (4H, t, *J* 4.9); **^{13}C NMR:** (125 MHz, $CDCl_3$) 163.27, 161.31, 157.21, 155.49, 140.54, 139.54, 136.67, 135.30, 133.50, 132.40, 128.67, 128.48, 128.42, 126.21, 115.76, 115.69, 115.52, 48.61, 45.31; **IR:** ν (cm^{-1}): 3304,1644, 1610, 1492, 1212, 823, 780; **HRMS:** $[M+H]^+$ calculated 477.1488, found 477.1504.

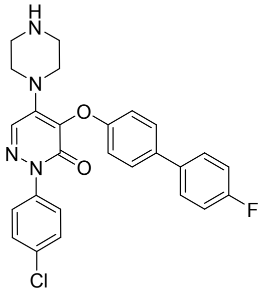

1.4. Human and asinine *ex vivo* tissue models

All human tissue was obtained with written informed patient consent and the study was approved by the Regional Ethics Committee. Human lung samples (patients aged 55-81 years) were obtained from the periphery of lung carcinoma resections (n=12) or from surgical biopsies for the investigation of interstitial lung disease (n=5). Fresh samples were placed immediately into transport medium consisting of saline 0.9%, 1% penicillin/streptomycin (Life Technologies, USA), 1% amphotericin B and 0.5% gentamicin (Sigma-Aldrich, USA) at 4°C. For fibre confocal fluorescence microscopy (FCFM) imaging, tissue was further dissected to approximately $2mm^3$ sections and placed in a 96 well plate. A section of tissue was also fixed in 10% buffered formalin for immunohistochemical analysis, while the remainder was homogenised in phosphate buffered saline (PBS) using a drill homogeniser.

Whole *ex vi*vo asinine lungs were collected from aged donkeys during routine necropsy at two UK donkey sanctuaries. Fibrotic lungs were selected because of grossly visible fibrosis. Following FCFM imaging, tissue samples were collected from each lung into 10% buffered formalin before undergoing routine processing to paraffin blocks. Further tissue was homogenised as above. As this study utilised only *ex vivo* tissue collected at routine necropsy, licensing on ethical and humane grounds was not required.

Tissue homogenates were evaluated with regard to protein retrieval using a BCA protein assay (Pierce™, Thermo Fisher Scientific Inc, Waltham, USA) with standard protocol. Homogenates (1mg/ml protein) were then pre-incubated with BAPN, pargyline, clorgyline, Inh-1 or Inh-2 in 25mM PIPES, 0.5% Triton X-100 (Sigma Aldrich, St Louis, MA, USA) for 1hr at 37˚C prior to addition of 10µM probe. The resultant fluorescence over a two hour time-frame was then measured using a Synergy™ H1 Hybrid Multi-Mode Microplate Reader (BioTek, Winooski, USA) at 37°C with filter excitation/emission 485/ 528nm respectively. Statistical analysis was carried out using Graphpad Prism 5 software, (GraphPad Software, Inc., San Diego, USA.) with statistical significance determined as $p<0.05$.

1.5. Western Blots

Tissue homogenates were prepared at standardised protein concentration (20-30µg/well) and electrophoresed on Bis-Tris 4-12% gradient gels (Invitrogen, Life Technologies, USA) before transfer to a nitrocellulose membrane. Membranes were blocked in 5% milk powder solution for 1h at room temperature then incubated overnight at 4°C with a 1:1000 dilution of either anti-LOX or anti-LOXL2 rabbit polyclonal antibodies (Abcam ab31238/ab96233, Cambridge, UK). Bands were visualised by chemiluminescence (SuperSignal® West Pico Chemiluminescent Substrate, Pierce™, Thermo Fisher Scientific Inc, Waltham, USA) and exposure on x-ray film. An anti β-Actin antibody (MAB1501, EMD Millipore, Merck KGaA , Darmstadt, Germany) was used to control for protein load.

1.6. Immunohistochemistry

LOX and LOXL2 were detected using the antibodies detailed above and goat anti-rabbit horseradish peroxidase-linked secondary antibody for diaminobenzidine detection (Bond refined staining kit, Leica Biosystems). A BONDTM immunostaining robot (Leica Biosystems, Germany) was used. An isotype control consisting of anti-GFP (A-11122, Invitrogen, Life Technologies, USA) was also performed.

1.7. Fibred confocal fluorescence microscopy (FCFM):

The FCFM system used was Cellvizio™ (Mauna Kea Technologies, Paris, France) encompassing a fibre-optic mini probe, laser scanning unit and image acquisition software. The 1.4mm diameter mini probe (ProFlexTM S-1500, Mauna Kea Technologies, Paris, France) is composed of a bundle of 30,000 cores and is 3 metres in length. It has a lateral resolution of 3.5 µm and depth of focus of 0-50µm. The fibre bundle connects to the laser scanning unit which contains a 488nm laser source.

Each individual core acts as a point source and point detector, transmitting the laser beam and collecting the returning fluorescent light emitted from the tissue. The system ensures the sequential injection of the laser beam into each fibre core and collects the returning fluorescent signal between 500nm and 650nm for imaging at a rate of 12 frames per second.

Whole asinine lungs were connected via a cuffed endotracheal tube to a positive pressure mechanical ventilator (Figure S3-4). A video endoscope was then used to navigate to a region of interest within the ex vivo lung. The endoscope was secured within a bronchus before the miniprobe was passed down the working channel of the endoscope and advanced into the alveolar space. 200µM oLOX was then instilled in a total volume of 1ml in PBS. Images were taken for for a minimum of 2min, giving

rise to over 1000 frames per video. Videos were taken at baseline for intrinsic pulmonary autofluorescence and at 15-30min following addition of probe.

Human tissue samples were incubated with buffer (25mM PIPES, 0.5% Triton X-100) +/- 1mM BAPN at 37°C for 1 hour prior to the addition of 10µM probe (total volume 60ul). The miniprobe was then directed over the surface of the tissue. Images were taken for each sample for a minimum of 60 seconds, giving rise to over 700 frames per video. Videos were taken at baseline for intrinsic pulmonary autofluorescence and at 60-90 minutes following addition of probe +/- BAPN.

Analysis of videos was by Cellvizio™ Viewer (Cellvizio™ Viewer 3.8.3, Mauna Kea Technologies, Paris, France) with a region of interest across the entire field of view (430 x 307 nm) for all frames and the mean frame fluorescence was calculated for each frame (giving >700 readings per video). Each individual experiment for probe +/- BAPN was corrected to the corresponding autofluorescence for that patient sample. The means (+/-SEM) for each experiment are reported and analysis was by a Mann Whitney u test by GraphPad Prism version 5.00 for Windows, GraphPad Software, San Diego California USA.

2. Supplementary Figures:

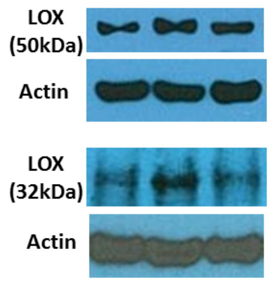

Figure S1: Mature (32kDa) LOX is expressed in human lung tissue homogenate. Western blot of LOX expression in aged human lung tissue. Strongest bands for LOX were around 50kDa, consistent with the glycosolated pro-lysyl oxidase, but mature 32kDa LOX was also present in several samples. Data representative of a minimum of 3 experimental replicates.

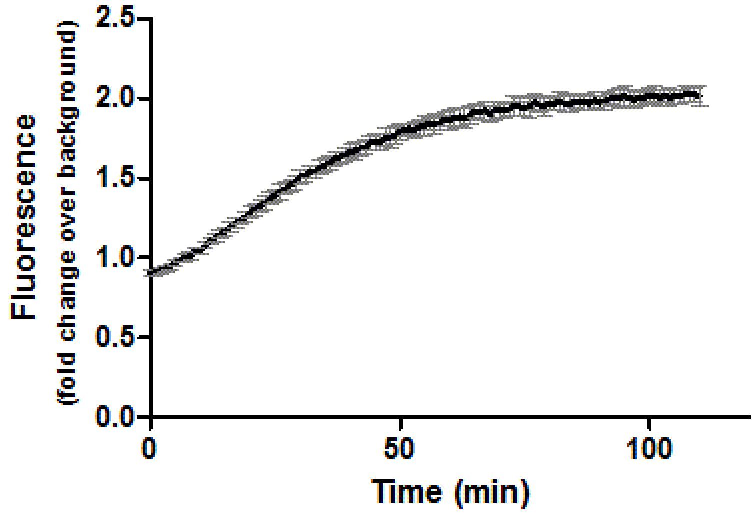

Figure S2: Kinetic study of fluorescent signal amplification obtained with oLOX in human tissue homogenate. Fluorescent signal generated by 10µM oLOX during a 2h incubation with human lung tissue homogenate is plotted as fold increase in fluorescence over background. A significant increase in fluorescent signal was obtained at 30min incubation ($p=0.0029$, one sample t test). Error bars represent SD, n=3.

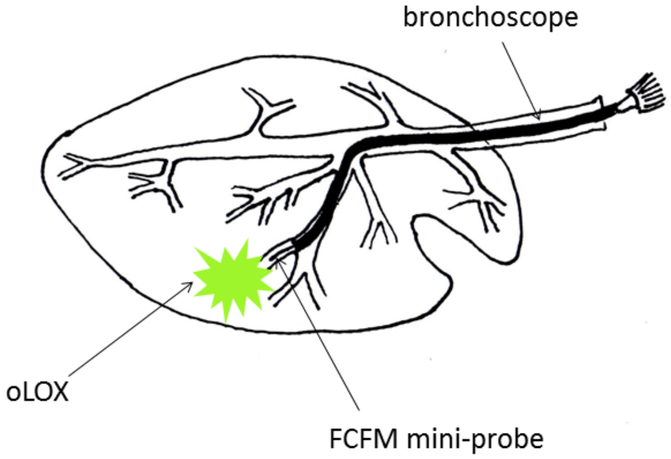

Figure S3: Schematic diagram of a mechanically ventilated *ex vivo* asinine lung as utilised in video figure S4.
